# Antimicrobial Stewardship: The Need to Cover All Bases

**DOI:** 10.3390/antibiotics2030400

**Published:** 2013-08-27

**Authors:** N. Deborah Friedman

**Affiliations:** 1 Barwon Health, Bellerine St, Geelong, VIC 3220, Australia; E-Mail: Deborahf@barwonhealth.org.au; Tel.: +1-61-4215-2033; Fax: +1-61-4215-2385; 2 Deakin University, Geelong, VIC 3216, Australia

**Keywords:** antimicrobial, stewardship, resistance, antibiotics

## Abstract

Increasing antimicrobial resistance has necessitated an approach to guide the use of antibiotics. The necessity to guide antimicrobial use via stewardship has never been more urgent. The decline in anti-infective innovation and the failure of currently available antimicrobials to treat some serious infections forces clinicians to change those behaviors that drive antimicrobial resistance. The majority of antimicrobial stewardship (AMS) programs function in acute-care hospitals, however, hospitals are only one setting where antibiotics are prescribed. Antimicrobial use is also high in residential aged care facilities and in the community. Prescribing in aged care is influenced by the fact that elderly residents have lowered immunity, are susceptible to infection and are frequently colonized with multi-resistant organisms. While in the community, prescribers are faced with public misconceptions about the effectiveness of antibiotics for many upper respiratory tract illnesses. AMS programs in all of these locations must be sustainable over a long period of time in order to be effective. A future with effective antimicrobials to treat bacterial infection will depend on AMS covering all of these bases. This review discusses AMS in acute care hospitals, aged care and the community and emphasizes that AMS is critical to patient safety and relies on government, clinician and community engagement.

## 1. Introduction

Antibiotic resistance is an important public health concern [1], which is emerging rapidly on a global scale [2,3,4]. This increase in antimicrobial resistance has necessitated an approach to guide the use of antibiotics [4]. This necessity has never been more urgent. The decline in anti-infective innovation and the failure of currently available antimicrobials to treat some serious infections is forcing clinicians to change those behaviors that drive antimicrobial resistance [5,6].

Antibiotic development has been dwindling since the 1990s. Compared with 1983 to 1987 when 16 new antimicrobials were developed, this number has steadily declined to a mere two new antimicrobials developed from 2008–2012 [7]. Furthermore, there are a limited number of new antimicrobial agents currently in development that have microbiological activity against multidrug-resistant gram-negatives [2,3,4].

According to the Infectious Diseases Society of America (IDSA), antimicrobial stewardship (AMS) includes not only limiting inappropriate use but also optimizing antimicrobial selection, dosing, route and duration of therapy to maximize clinical cure or prevention of infection while limiting unintended consequences, such as the emergence of resistance. The ultimate goal of antimicrobial stewardship is to improve patient care and health care outcomes [8]. AMS can and ought to be practiced in hospitals, long-term care facilities, and the community in order to effectively curb a public health crisis.

## 2. Locations for AMS Programs

### 2.1. Antimicrobial Stewardship in Hospitals

Hospital-based AMS programs are based on education coupled with either a “front-end” (or primary) restriction of antimicrobial availability or a “back-end” (or retrospective) review of broad-spectrum empirical therapy with advice regarding streamlining or discontinuing therapy, on the basis of culture and susceptibility testing results and clinical response [3,9]. Hospital-based AMS programs are typically staffed by an antimicrobial pharmacist who performs post-prescription ward rounds supported by an infectious diseases team [1].

There are several other partners that are critical in hospital-based AMS programs. Medical Microbiologists and the microbiology laboratory have an important and often leading role to play in AMS both inside and outside of working hours [10,11]. The microbiologist and laboratory staff frequently monitor blood cultures, dispense prescribing advice, and provide timely antibiotic susceptibility data to allow for rationalization of empiric prescribing [10]. In one study, hospitals that routinely reported susceptibility results for restricted antibiotics had significantly lower median total antibiotic use [10]. In addition, new organism detection techniques, such as rapid polymerase chain reaction and matrix-assisted laser desorption and ionization time-of-flight mass spectrometry allows microbiologists to also play a pivotal role in improving time to optimal therapy, and decreasing hospital length of stay and total costs [12,13]. Clinical pharmacists are also increasingly important partners to infectious diseases physicians in implementation of antimicrobial stewardship programs [14]. Antimicrobial pharmacists share responsibility for activities such as development of guidelines for antimicrobial use, education of physicians and other health care professionals, review of hospital antimicrobial orders with feedback to providers, administration of restrictive strategies, pharmacokinetic consultation, and research on program outcomes [15]. Hospital programs in the future should recruit other champions, such as nursing staff, to the cause of stewardship. Nursing staff also have the potential to make a large contribution to the management of antimicrobials within an in-patient setting as they have the most consistent presence as patient carers [16].

In hospital-based AMS programs that utilize more primary antimicrobial restriction to antibiotic availability, restrictions are placed on the availability of certain antimicrobial agents. Examples of such restrictions include the need for preapproval before the administration of restricted agents, and the use of special antimicrobial request or prescribing forms. Such programs, when properly implemented can result in large reductions in the overall use of some antimicrobials, but they are also time-consuming, labor intensive, require clinician buy-in and rely on experienced infectious diseases clinicians for their effectiveness [2,17].

The labor intensity of hospital-based AMS coupled with the rapid turnover of junior medical staff in many teaching hospitals create further challenges to both clinician engagement and AMS program sustainability in the longer-term. Furthermore, it may be that the prescribing practices of junior medical staff are not primarily influenced by guidelines. It has been demonstrated that the influences on prescribing behavior of non-consultant hospital medical staff are largely informal [18]. Prescribing by non-consultant medical staff in hospitals is based primarily on the immediate influence of more senior colleagues, with only a very minor influence from prescribing guidelines [18].

Aside from prescribing habits, there are several other factors that influence hospital-based prescribing. Clinicians are faced on a daily basis with patients whose outcomes will be markedly improved if treated early with broad-spectrum antibiotics [19,20]. Many hospital-based AMS programs do not primarily restrict antibiotic availability because of ethical concerns about the consequences of restriction of supply for acutely ill patients. The ethical conflict lies between the individual patient’s right to receive the “best” antibiotic and the need to decrease the future number of drug-resistant pathogens [21,22]. These concerns may be well founded and will remain given the increase in the use of immunosuppressive medications in patients residing in the community [23], and the well described increase in multi-resistant organisms in both hospitalized and community-based patients [3]. 

Hospital size will also influence AMS. Smaller hospitals or community-based hospitals may face major challenges developing and implementing AMS [24]. Storey *et al*. have described the implementation of an AMS program on the medical-surgical service of a 100-bed community hospital employing a core strategy of post-prescriptive audit with intervention and feedback. Their program, which had limited resources, was effective at reducing by 22% the defined daily doses of antimicrobials per 100 admissions [25]. Limited resources in another community institution influenced the implementation of a pharmacist-led AMS service using a clinical pharmacist and infectious disease physicians with the assistance of pharmacy residents and students [26]. Aside from the need for ward-based AMS in smaller regional or community hospitals, elective surgery is undertaken and substantial opportunities exist in smaller hospitals to improve compliance with published surgical antibiotic prophylaxis recommendations [27].

Prescribing is also influenced by ease and practicality. Both ease and practicality dictate that third-generation cephalosporins administered once daily are preferred over narrower spectrum penicillins (which require multiple daily doses or continuous infusion) in order to facilitate patient discharge in hospital-in-the-home programs. Ceftriaxone is a broad-spectrum cephalosporin with pharmacokinetic and antimicrobial characteristics that make it a good candidate for outpatient parenteral therapy [28,29]. Although anti-staphylococcal penicillins are the treatment of choice, studies have found that ceftriaxone compares favorably with oxacillin in the treatment of Methicillin-Susceptible *Staphylococcus aureus* infections [30]. Third-generation cephalosporins are however the driving force behind resistance in Gram-negative bacteria [31], and their restriction, while important in AMS programs, may result in reduced efficiency and higher costs to hospitals through increased length of inpatient stay in this setting.

In hospitals, constraints over the rapidity of completing patient assessment and care in emergency departments may also result in prescribing of broad-spectrum antimicrobials. The introduction of a 4-hour rule into tertiary hospital emergency departments necessitates efficient completion of patient assessment and treatment. This 4-hour rule resulted in fewer deaths and a significant fall in mortality for patients admitted through those emergency departments [32,33]. However, prescribing therapy for a variety of possible pathogens (without microbiological results) may negatively impact prescribing habits. More rapid point of care testing is needed in order to either inform or reduce antibiotic prescribing in this setting. 

Supporting hospital-based AMS is expensive and given that many antimicrobials which stewardship seeks to restrict are now off patent and inexpensive, there are no major cost-savings of restriction that will offset the costs of an AMS program [34,35,36]. However, it has been demonstrated that antibiotic-resistant infections (ARIs) are extremely costly [7,37]. Roberts *et al*. found that the medical costs attributable to ARI ranged from $18,588 to $29,069 per patient with an excess duration of hospital stay of 6.4–12.7 days, and an attributable mortality of 6.5% [37]. These substantial costs of ARI strongly argue for the potential economic benefits of resistance prevention programs.

### 2.2. Antimicrobial Stewardship in Long-Term Care Facilities

However, hospitals are only one location where antibiotics are used. Antimicrobial use is also high in residential aged care facilities where 2%–5% of people over the age of 65 years reside [38,39]. With the anticipation of a doubling in the number of adults older than 65 years by 2050, there will be an increasing need for beds in long-term care facilities (LTCFs) [40]. AMS must extend into post-acute and aged care facilities in order to have an impact. 

The elderly are more susceptible to infection due to transmission of infection within their residential environment, and primary and secondary immune dysfunction [41,42]. In addition, residents in LTCFs are frequently colonized with multi-resistant organisms (MROs) [43]. It is these infections that are the cause of death in many patients with any form of end-stage illness [44]. In addition, in non-acute facilities, withholding antibiotics may result in an increase in mortality rates. For example, among patients in an extended care facility, withholding therapy during febrile episodes was associated with significantly higher mortality rates when compared with patients who received antibiotics (59% *vs*. 9%) [21,45]. This type of evidence forms a compelling basis for prescribing of antibiotics in aged care. 

However, despite international guidelines suggesting the implementation of AMS in long-term care setting [46], well-delineated AMS strategies that are practical for this setting remain to be implemented on a large scale. In view of the differences in healthcare models and resources in aged care, AMS programs that function in the acute care hospital setting cannot be readily applied to the long-term care setting. It is thought that education and antimicrobial prescribing protocols in the aged care setting may help reduce unnecessary antimicrobial use [47].

Nicolle and others found that antimicrobials were the most commonly prescribed systemic drugs in long term care facilities and that up to three-quarters of antibiotic prescriptions for residents were inappropriate over a decade ago [48]. Since then, evidence has been mounting on both the importance and effectiveness of educational interventions and antimicrobial stewardship in LTCFs. 

It is known that antimicrobial prescribing in residential aged care is often not based on culture-proven infections, but is rather undertaken to manage reported symptoms, and most antimicrobial courses are initiated by telephone orders [49]. Recent studies reveal that antibiotics are prescribed for 65% of residents over seven months, and that such prescribing is inappropriate in 20%–80% of cases [50,51]. Research has shown that reducing antibiotic use causes a reduction in MDR Gram-negative bacteria and antimicrobial stewardship (including in LTCFs) is effective at reducing days of antibiotic therapy [52,53,54].

Jump and others have described their AMS program in a LTCF affiliated with a large urban Veterans Affairs (VA) medical center [55]. Their AMS team constituted an infectious diseases physician and a nurse practitioner who attended the facility weekly to deliver subspecialty expertise and were otherwise available for telephone consultation. Analysis revealed that 95% of their recommendations were followed, and that total antimicrobial use was reduced by 30%. After 18 months of AMS, they found statistically significant reductions in the use of both oral and intravenous antibiotics, including impressive reductions in fluoroquinolones, anti-pseudomonal cephalosporins and beta-lactam-beta-lactamase inhibitor combinations [43]. During this period, there was also a reduction in positive *Clostridium difficile* tests. In contrast, the authors do describe an increase in the use of ceftriaxone and carbapenems. In terms of the cost of this type of AMS service, it can be difficult to calculate as many teams who provide such services are full-time employees of affiliated institutions [55].

The widespread and inappropriate use of antimicrobials in the LTCFs is partly due to difficulties in clinically assessing elderly patients [56,57]. Other contributing factors include the absence of on-site microbiology laboratory and pharmacy services [58]. In addition, AMS can be difficult to enforce in aged care facilities because of multiple off-site service providers, in an environment where institutional antimicrobial prescribing protocols are lacking [59], and often a desire on the part of prescribers to treat residents before they become seriously unwell [56]. Stuart *et al*. studied the prevalence of antimicrobial organisms in aged care facilities and found that in the 15 months before the study commenced, residents received antibiotics for 445 episodes, 44% of which did not fulfill the McGeer criteria for bacterial infection [56,60]. They also found that 10 of 12 residents colonized with extended-spectrum β-lactamase (ESBL) producing organisms had received antibiotics within the previous 6 months. 

### 2.3. Antimicrobial Stewardship in the Community

Antimicrobial use in the community cannot be overlooked. Antibiotics are one of the most commonly prescribed drug classes worldwide, with considerable variation in outpatient antibiotic use between countries [61]. AMS in the community involves increasing public awareness, collection of antibiotic consumption and antimicrobial resistance data, and antibiotic conservation. Effective community-wide stewardship requires systems to be in place to monitor both antibiotic consumption and the appropriateness of prescriptions. However, in the absence of computerized prescribing, assessment of the quality of prescribing is more difficult to audit than consumption [62].

In Europe, the European Surveillance of Antibiotic consumption (ESAC) collects comprehensive data about antibiotic consumption, which allows benchmarking, and is complemented by longitudinal and compositional data analyses [63]. Data from 1997 through 2009 revealed a significant increase in total outpatient antibiotic use. They also found that the relative use of penicillins and quinolones significantly increased over time compared to sulphonamides and trimethoprim, and that there was significant seasonal variation, which decreased over time. Of the countries involved in ESAC, Greece had the highest antibiotic consumption, and Romania had the lowest [63].

Viral respiratory tract infections drive antibiotic overprescribing in the outpatient setting, and this overprescribing is also influenced by patient demand and expectations [64,65]. In keeping with this phenomenon of patient demand, IDSA’s call to action for the medical community in 2008, asked us to educate our patients [7]. Public misconceptions about the effectiveness of antibiotics are common. In a survey in the Netherlands, 47% of responders believed that antibiotics are effective against viral infections [66]. These beliefs need to be taken into account in developing AMS programs in the community. McCraig recognized in 2002 that educating parents about appropriate antimicrobial use is the single most important factor in reducing unnecessary antimicrobial use [67].

It has been shown that prescribing in the community can also be influenced by the doctor’s perception of parental expectations. A survey was conducted of 306 parents of children 2–10 years of age with the presenting complaints of ear pain, throat pain, cough, or congestion. The researchers found that physicians’ perceptions of parental expectations for antimicrobials were the only significant predictor of prescribing antimicrobials for conditions of presumed viral etiology. When physicians thought a parent wanted an antimicrobial, they prescribed them 62% of the time *versus* 7% of the time when they did not think the parent wanted antimicrobials for their child [68].

Community-wide AMS programs require governments to invest heavily in education for both prescribers and the “worried-well” in order to be effective. Advertising campaigns using all types of media forums have targeted raising awareness about the use of antibiotics. This has been exemplified well in Europe by national antibiotic awareness campaigns from the European Centre for Disease Prevention and Control (ECDC). Currently national campaigns take place in 29 European countries [69]. Slogans such as; “Unfortunately no amount of antibiotics will get rid of your cold” and, “Remember, antibiotics will not help your defenses against a cold” delivered the message about antibiotics to the public [69]. The ECDC also provides a toolkit for engaging in social media activities to promote prudent antibiotic use [70].

National campaigns are also underway in The United States of America, Canada and Australia. The United States Centers for Disease Control and Prevention’s campaign is entitled “Get Smart: Know when antibiotics work” [71]. The Canadian antibiotic awareness campaign and Australia’s NPS Medicinewise programs target both patients and prescribers [72,73]. Australia’s campaign engaged social media encouraging the public to join the fight against antibiotic resistance by becoming a “resistance fighter” [73].

In the 1990s, one of the first national campaigns to reduce antibiotic prescribing resulted in a decrease in antibiotic use and penicillin-resistant *Streptococcus pneumoniae* in Iceland [74]. Since that time, there have been many other public campaigns, with a gradual increase in published data about their impact. Good evidence correlating a national campaign to a reduction in antibiotic use has come from Belgium.

Strong interdisciplinary leadership can be an important asset in developing national campaigns to target antimicrobial resistance. In Belgium, the Belgian Antibiotic Policy Coordination Committee (BAPCOC) established working groups composed of microbiologists, infectious diseases’ and infection control specialists, epidemiologists, general practitioners (GPs), pharmacists, nurses, veterinarians, basic researchers, public health experts and health economists. Using their combined expertise, multimedia campaigns were developed to promote the prudent use of antibiotics in the community, and surveillance programs on antibiotic use and resistance were established [75]. Their mass media campaigns were associated with a 36% reduction in antibiotic prescriptions between 1999–2000 and 2006–2007 [75]. In addition, over a 5-year period, the Belgian campaign was able to show a substantial reduction in macrolide resistance in *Streptococcus pyogenes*. 

There is now increasing evidence that community-based AMS of a variety of agents can result in improvements in antimicrobial resistance. Gottesman *et al*. evaluated the impact of quinolone restriction on the antimicrobial resistance of *E. coli* urine isolates in a community setting [76]. They assessed the proportion of quinolone-susceptible *E. coli* urine isolates in the periods before, during, and after implementation of a nationwide restriction on ciprofloxacin use. The restriction campaign resulted in a significant decrease in *E. coli* nonsusceptibility to quinolones, from a mean of 12% before the intervention period to a mean of 9% in the intervention period (odds ratio, 1.35; *p* = 0.014). They were also able to demonstrate that the reversal in antimicrobial resistance may be short-lived as the improved susceptibility pattern reversed immediately when quinolone consumption rose [61].

France used to be known for the highest rates of antibiotic use and pneumococcal resistance in Europe. In 2001, French policy makers and public health authorities developed a coordinated and multifaceted strategy. It involved a yearly campaign targeting the public via mass media, conveying the message that “Antibiotics Are Not Automatic” (especially for viral respiratory tract infections). In addition, they promoted both treatment guidelines and the use of the Streptococcal rapid antigen test. Primary care physicians were also targeted by one-on-one educational sessions (referred to as academic detailing). Using 90% complete national health insurance data for two winters before the campaign and five winters after the launch, they measured a decline of 26.5% in the number of antibiotic prescriptions. This exceeded their national target of a 25% reduction in prescribing. They also found that the greatest decrease in prescribing was observed in children and that the use of all antibiotic classes except fluoroquinolones decreased. The relative increase in fluoroquinolone use by 12.8% was identified and the researchers hypothesized that this may be explained by the choice of antibiotic becoming less appropriate for certain treatment indications [77].

Aside from measuring antibiotic use and antimicrobial resistance, a multidisciplinary program in Sweden has been able to show that antibiotic use can be reduced without measurable negative consequences. Swedish Strategic Programme for the Rational Use of Antimicrobial Agents and Surveillance of Resistance (STRAMA) was initiated in Sweden in 1994 with a focus on inappropriate prescribing of antibiotics to children with respiratory tract infections and on the surveillance of resistance in pneumococci. Between 1993 and 1997 antibiotic prescribing was reduced by 22%, with the most pronounced reduction in children under six years of age. In addition, the national frequency of penicillin-non-susceptible pneumococci did not increase during the 1990s [78]. STRAMA was also able to show a 20% decrease in antibiotic use for outpatients, and a 52% reduction in antibiotic use in children aged 5–14 with no increase in hospital admissions for acute mastoiditis, rhinosinusitis, and quinsy (peritonsillar abscess) [79].

National antimicrobial stewardship campaigns are expensive however. In France, developing and conducting their campaign cost 500 million EUR (653 million USD) over six years. In Belgium, the program cost 400,000 EUR (523,000 USD) per year, and in 2007 their annual budget was 7.8 million EUR (10.2 million USD). Without a more detailed cost-benefit analysis, this cost information is difficult to interpret. However, it is known that the reduction in antibiotic costs in France outweighed the cost of the public campaign to reduce prescribing [80].

An assessment of cost issues relevant to national AMS programs must take into account the amount of money spent by the pharmaceutical industry to promote antibiotics. In the United States in 1998, it was estimated that pharmaceutical companies spent about US $1.6 billion to promote antibiotics, an amount that vastly exceeds any budget allocated to antibiotic restriction campaigns [81].

Some of the more innovative campaigns aimed at reducing antibiotic use have used different outcome measures as their marker of success. The ideal outcome measure of antibiotic use is the incidence of *Clostridium difficile* infection (CDI). In Scotland, their program aimed to reduce the use of “*C. diffogenic* agents”, termed the “4 C’s antibiotics”. The antibiotics that they targeted for reduction were; cephalosporins, amoxicillin-clavulanic acid, clindamycin and ciprofloxacin, and they promoted the use of amoxicillin, co-trimoxazole, doxycycline, and gentamicin. Compared with 2008, there were 47,000 fewer antibiotic prescriptions, and the use of the 4 C’s decreased by 30%. There was also a 5% increase in the use of recommended antimicrobials over the study period. They were also able to demonstrate an impressive 42% reduction in the rate of CDI cases from 2006 to 2010 [82].

Antibiotic delay is a strategy that is used in the community and indeed should be encouraged. More use should be made of collecting samples for culture when it is thought that a prescription is justified. The majority of patients in general practice will not come to harm if a prescription is delayed overnight to allow time for initial culture, which may direct treatment [83].

Other strategies for better using existing antibiotics aim to optimize dosing, scheduling and duration. There is evidence of the influence of antibiotic duration on minimizing the development of antibiotic resistance. Drusano *et al*. describe instances when drug dose and/or schedule can be used to minimize the probability that mutants will take over the bacterial population [84]. In addition, Mouton *et al*. describe the concept of antibiotic conservation, which involves the optimization of dosing regimens by choosing the dose and schedule that results in the antimicrobial exposure that will achieve the microbiological and clinical outcome desired while simultaneously suppressing emergence of resistance [85]. The authors also suggest that both pharmacokinetic and pharmacodynamic relationships of older antibiotics need to be urgently established in order to inform the best ways to use old drugs [85].

## 3. Impacts of Antimicrobial Stewardship

A reduction in total antibiotic use and expenditure are impacts of AMS, which have been well demonstrated [25,74,75,77,79,86,87,88]. AMS has also been shown to improve patient outcomes, and minimize antimicrobial resistance [74,75,76,89]. The Scottish Antimicrobial Prescribing Group has demonstrated not only a significant reduction in *Clostridium difficile* infection rates in Scotland, but their program has also been successful at improving the clinical management of infections through quality improvement methodology [82]. Furthermore, AMS programs promote review of physician orders and therefore have been shown to allow for early detection and intervention of prescription errors [79]. Quality of care indicators, including patient safety are also improved secondary to AMS, and AMS can reduce hospital length of stay [88,90]. 

## 4. The Role of Health Care Information Technology

Traditionally, AMS programs relied on manual methods combined with clinical oversight and intervention. However, the advent of improved modern health care information technology has offered the opportunity to expand the breadth, depth, and efficiency of AMS programs. Point-of-care access to current medical information is easily available to the practitioner through the use of smartphones, iPads, and other personal digital assistants [91]. In addition, mobile health has enormous scope within AMS to both assist patients with antibiotic reminders, and to assist busy clinicians with antimicrobial information, clinical prescribing support and efficient collection of antimicrobial data [92,93]. 

Expert computer clinical decision support systems have been a very promising information technology advance [94]. A seven-year study conducted in Salt Lake City, Utah, implemented computer-assisted decision support programs using local clinician-derived practice guidelines. They demonstrated that such decision support could improve antibiotic use, reduce associated costs, and stabilize the emergence of antibiotic-resistant pathogens in their institution [95]. A computerized antimicrobial approval system developed in Australia has also shown effectiveness in reducing the use of both 3rd and 4th generation cephalosporins, fluoroquinolones, glycopeptides and carbapenems with a favorable influence on local antibiograms [86,87]. Electronic clinical decision support via handheld devices has been shown to significantly reduce patient length of stay and antibiotic prescribing in a critical care unit, and to improve the appropriateness of antimicrobial selection for acute respiratory tract infections in a rural primary care setting [90,96]. 

Recently, researchers in Minneapolis compared the appropriateness of antimicrobial courses ordered either with their computer decision support system or without such a system. They found that use of a decision support system was associated with significantly more appropriate antimicrobial prescribing [97]. In addition, a meta-analysis of studies found that clinicians with decision support were 1.6 times more likely to order recommended treatments than providers without such a system [98]. 

However, this optimism must be balanced against potential pitfalls of electronic systems used in health care. Electronic health records are widely used in the United States, but many of these do not meet criteria for “meaningful use” [99,100]. Only a small number of institutions have been able to demonstrate an improvement in safety, quality and efficiency as a result of such electronic systems [100]. In addition, an evaluation of a computerized decision support system (TheradoC) in Nebraska, which triggered prospective alerts regarding both the patient’s condition and the antimicrobial therapy, revealed that over 8,000 alerts were received over a 5-month period [101]. Only 30% of these alerts were actionable, and the labor intensity of reviewing these alerts was substantial at approximately 5 hours per day. This type of system resulted in alert fatigue by staff [101]. Moreover, these types of systems need to move beyond the pre-prescription phase, and support the post prescription review process in order to be effective.

The dissemination of well-constructed electronic decision support systems has not been broad to date, likely due to issues of cost, the requirement for compatibility with existing electronic systems, and the fact that such systems cannot stand-alone and be effective. Such technology to assist prescribing must be part of a broader framework of AMS with an engaged AMS team in order to have an impact. 

## 5. Rapid Diagnostic Testing

Rapid diagnostic testing (RDT), although not yet routine, is an important investment, which will likely contribute to a reduction in antibiotic use. RDT allows for rapid identification of group A streptococcus, human immunodeficiency virus (HIV), and bacteria such as methicillin-resistant *Staphylococcus aureus* (MRSA), *Clostridium difficile*, and extended spectrum beta-lactamase (ESBL)-producing organisms [102,103,104,105,106,107,108].

Rapid diagnostics are still predominantly performed in a laboratory. Such tests include; direct microscopy of specimens; antigen detection tests (such as enzyme immunoassay); molecular detection (nucleic acid probes and nucleic acid amplification); rapid biochemical tests (such as nitrite and leukocyte esterase performed on a urine dipstick); and serologic testing [102].

Rapid and accurate diagnosis of an infection should enhance patient outcome by enabling early initiation of appropriate therapy and implementation of relevant infection control measures, and reducing unnecessary antimicrobial treatment. In particular, molecular tests such as multiplex-PCR or a DNA-based microarray platform for detection of organisms causing sepsis have been developed and hold promise for rapid diagnosis of bacterial sepsis [102,104]. Despite the rapid progress of RDT technologies, these diagnostic modalities have not yet made many inroads toward replacing standard identification tests in medical microbiology laboratories. The unavailability of certain rapid tests is typically blamed on prohibitive costs and their doubtful cost-effectiveness. In addition, molecular-based RDTs have relatively lower sensitivities or positive predictive values compared with traditional methodologies for investigation or diagnosis of infections [102].

Clinicians can also utilize serum markers in order to decide whether antibiotics ought to be prescribed in a given patient. C-Reactive protein (CRP) is an acute-phase reactant, and CRP level measurements are frequently used to aid in the diagnosis of bacterial infections [109]. In addition, procalcitonin (PCT) is a hormone that has emerged as a promising marker for the diagnosis of bacterial infections and may be used to support clinical decision making for the initiation and discontinuation of antibiotic therapy [110,111]. It has been shown that PCT level is more sensitive and specific than the CRP level for differentiating bacterial from either viral or non-infective causes of inflammation [109]. Studies have demonstrated that higher levels of PCT are found in severe bacterial infections than in viral infections and nonspecific inflammatory diseases [112]. Studies in patients with acute respiratory infections have shown that measuring PCT is effective in reducing antibiotic exposure and that PCT guidance was not associated with increased mortality or treatment failure in any clinical setting [110]. In addition, randomized controlled trials have demonstrated the feasibility of using PCT in settings ranging from primary care to emergency departments, hospital wards and intensive care units [113,114,115,116]. Further validation of the use of PCT in all age groups and in patients with a variety of comorbidities such as immune compromise will be informative for clinicians.

## 6. Conclusions

Preventing, reducing, and controlling the emergence of antimicrobial-resistant organisms is a major public health challenge that requires the participation of the entire medical community and public health agencies [18]. AMS initiatives should focus on the strategy of optimizing the use of currently available antimicrobials by using pharmacokinetic and pharmacodynamic principles such as extending time above the minimum inhibitory concentration in order to reduce antimicrobial resistance. It is however sobering to acknowledge that AMS in any setting, and the use of mobile health and RDTs may not be feasible for some time in resource-poor settings where MROs continue to evolve. AMS is critical to patient safety and relies on clinician and community engagement, and moving forward must routinely cover all bases. This requires government backing and strategies such as rapid diagnostics at the point of care and ongoing use of media forums to influence public opinion. 
